# Elucidating the Effect of Nutritional Imbalances of N and K on the Infection of *Verticillium dahliae* in Olive

**DOI:** 10.3390/jof8020139

**Published:** 2022-01-29

**Authors:** Ana López-Moral, Carlos Agustí-Brisach, Cristina Ruiz-Blancas, Begoña I. Antón-Domínguez, Esteban Alcántara, Antonio Trapero

**Affiliations:** Departamento de Agronomía, María de Maeztu Unit of Excellence 2020-23, Campus de Rabanales, Universidad de Córdoba, Edif. C4, 14071 Córdoba, Spain; b92lomoa@uco.es (A.L.-M.); cagusti@uco.es (C.A.-B.); cristinaruizblancas@gmail.com (C.R.-B.); z72andob@uco.es (B.I.A.-D.); ag1alvae@uco.es (E.A.)

**Keywords:** mineral nutrition, nitrogen, *Olea europaea*, potassium, Verticillium wilt

## Abstract

The effect of mineral nutrition on wilt diseases has been previously reported in many herbaceous hosts, though such an effect on Verticillium wilt in olive (*Olea europaea* L.; VWO), caused by *Verticillium dahliae*, is still uncertain. Field observations reveal that nitrogen (N) excess or imbalances of N-potassium (K) favour VWO epidemics. However, this has yet to be demonstrated. Thus, the aim of this study was to evaluate the influences of nutritional imbalances of N and K in *V. dahliae* infection of olive. To this end, adjusted treatments with N excess (↑N+↑Na), K deficiency (↓K) and their combination (↑N+↑Na+↓K) were evaluated on the viability of *V. dahliae* microsclerotia (MS), as well as on disease development in olive plants. In parallel, the potential indirect effect of the treatments on the viability of conidia and MS of *V. dahliae* was evaluated through the stimuli of root exudates. Treatments ↑N+↑Na and ↑N+↑Na+↓K decreased MS germination and disease progress, whereas ↓K significantly increased both parameters. Root exudates from treated plants increased the conidia germination of *V. dahliae* but reduced the MS germination. The results of this study will be the basis for planning further research towards a better understanding of the effect of mineral nutrition on VWO.

## 1. Introduction

The effect of mineral nutrition on Verticillium wilt diseases has already been reported in many herbaceous hosts, resulting in either a decrease or an increase in wilt symptoms [1]. However, there is a substantial lack of knowledge on the influence of mineral nutrition in the development of Verticillium wilt of olive (VWO; *Olea europaea* L.), which is considered the most serious disease of this woody crop in the Mediterranean basin [2,3,4]. It is well known that VWO is caused by the soil-borne fungus *Verticillium dahliae* Kleb., which is able to survive for a long time in the soil through the formation of microsclerotia (MS). MS not only serve as resistance structures, but are also the primary inoculum of the pathogen. They germinate due to the biochemical stimuli from host root exudates, giving rise to infectious hyphae that penetrate the roots of the plants and grow into the xylem vessels [1,5]. The biology of the pathogen suggests that important attention should be paid to elucidating the effect of soil mineral nutrition into its life cycle in olive tree, and particularly in the infection process in this woody host.

Regarding our knowledge of the effect of mineral nutrition on VWO, in a first approach based on field observations in the Guadalquivir valley (Andalusia region, southern Spain), it has been observed that excess nitrogen (N) or imbalances of N-potassium (K) favour VWO epidemics in affected olive groves [6,7]. However, despite this evidence, studies on the effect of plant mineral nutrition on the occurrence and development of VWO are almost non-existent. To the best of our knowledge, only Pérez-Rodríguez et al. [8] evaluated the interaction between irrigation and mineral nutrition on VWO in the susceptible olive cv. ‘Picual’ under semi-controlled conditions. These authors demonstrated that increased frequency of irrigation combined with fertilization of N-P-K (15-15-15) favoured the development of the disease in soils naturally infested by *V. dahliae*. However, in this study, no conclusions could be drawn regarding the effects of certain nutrients, such as N excess, K deficiency or the imbalances of N/K in VWO.

With regard to plant nutrition, it is generally understood that N influences a wide range of pathogens and their hosts, with the form of N available to the pathogen being essential for disease development, regardless of the rate of N available. The influence of N in plant diseases is due both to direct effects, such as alterations in the growth or virulence of the pathogen, and to indirect effects, such as changes in the physiology of the plant or in the biotic and abiotic environments, mainly in the rhizosphere since the pathogen is soil-borne [1,9,10]. The effect of N on *V. dahliae* infection has been previously reported in many herbaceous hosts, but with contradictory results in the literature. For instance, although the general understanding is that N excess increases disease incidence [1,9], direct effects of NO_3_^−^ and NH_4_^+^ have been reported by reducing the number of propagules of *V.* *dahliae* in the soil and consequently mitigating the disease [1]. 

Concerning the effect of K on plant diseases, it has also been reported that K alone, or in combination with N or other nutrients, influences the severity of diseases caused by many soil-borne pathogens, including Verticillium wilt diseases [1,11,12,13,14]. Indeed, applications of K in K-deficient soils increase host resistance against diseases such as Verticillium wilt of cotton [1]; nevertheless, the mechanisms implicated are still poorly understood [11,13]. Moreover, although there is a vast amount of literature on the relationship between K and plant diseases, the understanding of the relationship between K and other nutrients with plant diseases in agroecological ecosystems has been very little studied [11].

Therefore, determining the influence of nutritional imbalances of one or several nutrients on the incidence and severity of VWO is essential to establish an adequate nutrient program preventing major *V. dahliae* infections. This issue is even more relevant in the case of VWO since adequate nutrition management must be taken into account within an integrated disease management strategy [2,4,5]. In addition, this last aspect is of major importance to be considered in olive groves under fertigation systems, where not only the imbalanced mineral nutrition, but also the water regimes, can affect the severity of VWO [15,16], while the interaction between irrigation and fertilization can act in favour of VWO epidemics [8]. 

Therefore, as little attention has been previously given to understanding the relationship between N and K and other nutrients on VWO, the aim of this study was to evaluate the influences of nutritional imbalances of N and K, alone or combined, in *V. dahliae* infection to olive under laboratory-controlled conditions. To this end, adjusted treatments with N excess, K deficiency and their combination were tested on the viability of *V. dahliae* microsclerotia (MS) *in vitro* and on disease development by means of bioassays in olive plants of cv. Picual. In parallel, root exudates from treated plants were collected, evaluating the potential indirect effect of the treatments through root exudate stimuli on the viability of conidia and the MS of *V.* *dahliae* *in vitro*.

## 2. Materials and Methods

### 2.1. Fungal Isolate and Inoculum Preparation

The *V. dahliae* isolate V323, recovered from soil samples collected from a commercial olive orchard affected by VWO in Villanueva de la Reina (Jaén province, southern Spain), was used in all of the experiments in this study. Previously, it was characterized as the defoliating (D) pathotype by PCR following the protocol described by Mercado-Blanco et al. [17] and as highly virulent in olive plants by pathogenicity tests. It is maintained as a single-spore isolate on potato dextrose agar (PDA; Difco^®^ Laboratories, Maryland, USA) slants fully filled with sterile paraffin oil at 4 °C in darkness in the collection of the Department of Agronomy at the University of Córdoba (DAUCO, Córdoba, Spain). 

Prior to conducting the experiments, fresh colonies of the isolate V323 were obtained from the collection by plating small mycelial fragments of the colonized agar from the tube onto PDA acidified with lactic acid (APDA; 2.5 mL L^−^^1^ of medium) and incubated at 24 °C in the dark for 7 days. Subsequently, fresh colonies were transferred to PDA and incubated as described before. 

For inoculum preparation, 1 kg of cornmeal–sand mixture (CMS; sand, cornmeal and distilled water; 9:1:2, *w*:*w*:*v*) was placed in 2 L Erlenmeyer flasks. Subsequently, the flasks were sterilized following the protocol described by López-Moral et al. [5]. For CMS pathogen colonization, 50 mycelial plugs (7.5 mm in diameter) of *V. dahliae* isolate V323 growing on PDA were introduced into each flask. The flasks were incubated at 24 °C in the dark for 4 weeks and manually shaken once a week to favour a homogeneous colonization. Finally, the inoculum density of the colonized CMS was estimated by means of the serial dilution method on PDA and expressed as colony-forming units (CFUs) [5,18].

### 2.2. Treatments 

Five treatments were designed, containing different concentrations of N, K and Na (Table 1). The first treatment (Control N-K) contained moderate concentrations of N and K and was used as reference for the following treatments. The second treatment (↑N+↑Na) was designed to contain a high concentration of N, and because NaNO_3_ was used, it also contained a high concentration of Na. The third treatment (↑NaCl) contained a high concentration of Na as NaCl but not high N, in contrast with the second treatment. The fourth treatment (↓K) was similar to the Control N-K treatment but without K. The fifth treatment (↑N+↑Na+↓K) was similar to the second treatment but without K. All of the treatments were prepared by diluting the mineral nutrients (Merck KGaA, Darmstadt, Germany) in deionized distilled water (DDW). Thus, an additional treatment (Water) containing only DDW was included for comparative purposes.

### 2.3. Viability of Verticillium dahliae Microsclerotia

#### 2.3.1. Preparation of Artificially Pathogen-Colonized Substrate

The CMS colonized by *V. dahliae* isolate V323 was removed from the flasks, deposited in aluminium trays and air-dried on a laboratory bench at 22 ± 2 °C for 10–14 days. Subsequently, it was sieved with a 0.8 mm sieve, and the inoculum density of *V. dahliae*, expressed as the number microsclerotia (MS) per g of substrate, was estimated by the wet sieving method [19] using 10 replicated Petri dishes of modified sodium polypectate agar medium (MSPA) [20] following the protocol described by Varo et al. [21]. Due to the high MS concentration obtained (≈1300 MS per g of substrate), an adjusted amount of sterile sand was added to the colonized CMS to reduce the MS concentration to 200 MS per g of substrate (CMS-200). This step was necessary to homogenize the amount of inoculum and to make it possible to count the germinated MS in downstream analyses.

#### 2.3.2. Application of Treatments

The experiment was conducted using sterile transparent PVC pots (100 mL vol.) with holes in the base (5 holes, each 2 mm in diameter) to facilitate percolation. Subsequently, each pot was filled with 60 g of the CMS-200 and irrigated with 30 mL of the corresponding treatment. After the percolation, the pots were hermetically closed and incubated in an environmental controlled chamber at 22 ± 2 °C at 60% relative humidity (RH) for 1, 7 or 14 days (exposure periods). A completely randomized design was used with three replicated pots per treatment and different period of exposure combinations (6 treatments × 3 exposure periods × 3 replicated pots = 54 pots in total). The experiment was conducted twice.

#### 2.3.3. Assessment

After each exposure period, the pot contents were deposited in individual aluminium trays and air-dried in a vertical laminar flow cabinet at 22 ± 2 °C for 10–14 days. The inoculum density was estimated by wet sieving as described above, and it was expressed as the number of MS per g of CMS-200 (MSG). 

### 2.4. Verticillium Wilt Development

#### 2.4.1. Plant Material

Healthy 6-month-old rooted olive cuttings of cv. Picual (highly susceptible to *V. dahliae* [22]) growing in peat moss in opaque PVC pots (0.5 L) were obtained from a commercial nursery. To induce the active growth of the plants, they were pre-conditioned in a controlled-growth chamber at 22 ± 2 °C, with a 14:10 h (light:dark) photoperiod of white fluorescent light (10,000 lux) and 60% RH for 1 month. During this month, plants were irrigated three times per week using 350 mL of DDW per plant. 

#### 2.4.2. Application of Treatments and Plant Inoculation

After the preconditioning period of the plants, the last well-developed leaf on the stem was marked to estimate the effect of the treatments on plant growth. From this moment, plants were irrigated with the different treatments using 350 mL per plant once a week throughout the experiment. Moreover, plants were irrigated with 350 mL of DDW twice a week.

The plant inoculation was carried out one month after the first application of treatments. For this purpose, plants were transplanted to opaque PVC pots (0.8 L) previously disinfested with a commercial sodium hypochlorite solution at 20% for 2 h and filled with a 20% (*w*:*w*) mixture of colonized CMS and sterile sand (theoretical inoculum density of the final substrate = 10^7^ CFU g^−^^1^; [5,18]). Non-treated and inoculated plants were also included as a positive control. Additionally, plants that were transplanted in a similar way but with sterile CMS served as negative controls (non-inoculated plants). Just after inoculation, all plants were irrigated and incubated in a controlled-growth chamber at 20 °C in the dark and 100% RH for 7 days. Subsequently, light and humidity parameters were progressively modified over 1 week until they reached 23 °C, a 12 h photoperiod of fluorescent light (10,000 lux) and 70% RH, which were maintained until the end of the experiment (20 weeks after the first treatment application; 16 weeks after inoculation). 

A randomized complete block design (three blocks) was used with five treatments, with inoculated or non-inoculated plants as the independent variable and four replicated olive plants per treatment and block (6 treatments or control × 2 inoculation conditions (inoculated and non-inoculated plants) × 3 blocks × 4 replicated plants = 144 plants in total). The experiment was conducted twice.

#### 2.4.3. Assessment

Disease severity (*DS*) was evaluated weekly for 16 weeks after inoculation using a 0 to 4 rating scale with 17 values, which estimates the percentage of affected tissue by means of five main categories (0 = no symptoms; 1 = 25%, 2 = 50%, 3 = 75%, and 4 = 100%, dead plant) of affected tissue, with three intermediate values (0.25, 0.50 and 0.75) between the main categories [5,23]. Therefore, the scale values (*X*) were related to the percentage of affected plant (*Y*) according to the equation: *Y* = 25*X*. At the end of the experiment, *DS* data were used to calculate the area under the disease progress curve (*AUDPC*) using the following formula:

(1) $$AUDPC=\overset{n}{\underset{i=1}{\sum }}[(DS_{i+1}+DS_{i})/2](t_{i+1}\times t_{i})]\;$$

where *DS* is the scale value described above at each evaluation moment (*i*), *t_i_* is the time (days) at the evaluation moment, and *n* is the total of number of evaluations [24]. Subsequently, both the final *DS* and *AUDPC* were expressed as relative percentage (RDS, RAUDPC; %) to disease parameter values of the inoculated plants treated with only DDW (Water (+)) at the end of the experiment. In addition, disease incidence (DI) and mortality were assessed as the percentage (%) of symptomatic or dead plants, respectively. At the end of the experiment, the apical shoot growth was determined by the length increase and its fresh weight after the 20 weeks of treatments. 

Finally, one plant per block and treatment of inoculated and non-inoculated plants was randomly selected to establish the infection by means of fungal re-isolation from the stem. For this purpose, basal stems of the selected plants were washed under running tap water for 2 h. Subsequently, small fragments of the asymptomatic or affected tissue were cut and surface-sterilized by dipping them in a 10% solution of commercial bleach (Cl at 50 g L^−^^1^) for 1 min, air-dried on sterilized filter paper for 10 min, and plated onto APDA. There were three Petri dishes per plant, with seven attempts at isolation (fragments of stem). Petri dishes were incubated at 24 °C in the dark for 7 days. The consistency of isolation was estimated as a percentage (%) of positive attempts.

### 2.5. Effect of Root Exudates from Non-Inoculated Plants Grown under Different Treatments

#### 2.5.1. Collection of Root Exudates

One plant per each block of the treated and non-inoculated plants (*n* = 6) was randomly selected to collect root exudates. To this end, plants were taken out of the pots, and the substrate was carefully removed from the roots by spraying tap water until most of the substrate was removed. Finally, the roots were washed by dipping them into DDW. Immediately after washing, intact plants were placed individually in cylindrical opaque PVC pots (9.5 cm in diameter × 11 cm in height; ≈0.5 L vol.) without drainage holes and containing 350 mL of 0.01 M CaSO_4_ × 2 H_2_O [25,26] with the complete root system submerged. For the fixation of the plants, polyethylene discs 10 mm in diameter were designed, with a small hole (4 mm in diameter) in the centre to fix the basal end of the stem. Then, the pot was closed with the polyethylene disc with the fixed plant, keeping the entire root system in the collection media, and the pots were incubated for 4 h at room temperature in the dark. Subsequently, the content of each pot was recovered (CaSO_4_ + root exudate solution) and double filtered, first through filter paper (43–48 µm pore size; Filter-Lab^®^, Filtros Anoia, Barcelona, Spain) to remove root detritus, and then through a 0.45-μm membrane filter to remove microbial cell debris. The filtrate was immediately frozen in plastic vials at −20 °C until further analyses were conducted. Prior to use, the vials were defrosted overnight at 4 °C in the dark. The root-exudate collection method from olive plants was adapted by López-Moral et al. [26] from previous protocols described by Aulakh et al. [25].

#### 2.5.2. Viability of *Verticillium dahliae* Microsclerotia

Soil samples from a commercial cotton (*Gossypium hirsutum* L.) field naturally infested with *V. dahliae* were collected (Villanueva de la Reina; Jaén province, Spain; Geographic coordinates 38°00′10.8″ N 3°55′57.5″ W). Sampling and laboratory processing were conducted as described by López-Moral et al. [5].

Sterile transparent PVC pots (100 mL vol.) with holes in the base were prepared and filled with 60 g of the naturally infested soil. Subsequently, each pot was irrigated with 30 mL of the collected root exudates. Additionally, pots filled with 60 g of naturally infested soil irrigated with 30 mL of CaSO_4_ collecting solution without root exudate were included as a control. After the percolation, the pots were hermetically closed and incubated at 22 ± 2 °C for 72 h in the dark. A completely randomized design was used with three replicated pots per root exudate collected from each of the three selected plants per treatment (*n* = 6) or control (*n* = 1). The experiment was conducted twice.

After incubation, the treated soil samples were removed from the pots, and they were then processed to estimate the inoculum density of *V. dahliae* as described in Section 2.3.1.

#### 2.5.3. Viability of *Verticillium dahliae* Conidia

Conidial suspensions were obtained from 14-day-old colonies of *V. dahliae* isolate V323 growing on PDA as described before and were adjusted to 8 × 10^5^ conidia mL^−^^1^ using a haematocytometer. A 5 μL drop of the conidial suspension was placed in the centre of a microscope coverslip (20 × 20 mm); subsequently, a 5 μL drop of the root-exudate solution was mixed. Coverslips were placed inside Petri dishes containing water agar, which were used as humid chambers, and incubated at 23 ± 2 °C in the dark for 24 h. A completely randomized design was used with three replicated coverslips per root exudate collected from each of the three selected plants per treatment or control (*n* = 7). The experiment was conducted twice.

After the incubation period, a 5 µL drop of 0.01% acid fuchsine in lactoglycerol (1:2:1 lactic acid:glycerol:water) was added to each coverslip to stop conidial germination, and the coverslips were mounted on slides. A total of 100 randomly selected conidia per coverslip were observed at ×400 magnification using a Nikon Eclipse 80i microscope (Nikon Corp., Tokyo, Japan), and germinated and non-germinated conidia were counted. Conidia were considered germinated when the germ tube was at least one-half of the longitudinal axis of the conidia. Conidial viability was estimated as the percentage (%) of germinated conidia (CG) (*n* = 7; 6 treatments and control). 

### 2.6. Data Analyses

Data from the two repetitions of each experiment were combined after checking for homogeneity of the experimental error variances by the F test (*P* ≥ 0.05). Subsequently, in all cases, data were tested for normality, homogeneity of variances, and residual patterns, and they were logarithmically transformed when necessary. To determine the effect of treatments on MS viability, a factorial ANOVA was previously conducted with ‘MSG’ as the dependent variable and ‘treatments’, ‘exposure periods’, and their interaction as independent variables. Because the interaction was significant (*P* < 0.05), independent ANOVAs were conducted to study the effect of exposure periods on MS viability for each treatment, as well as the effect of treatments on MS viability for each exposure period. For the experiment *in planta*, two one-way ANOVAs were conducted with the ‘RAUDPC’ or ‘RDS’ as the dependent variables and the ‘treatment’ as the independent variable. RAUDPC and RDS were compared according to Fisher’s protected LSD test at *P* = 0.05 [27]. Data on the final DI (% of affected plants) and mortality (% of dead plants) were analysed by multiple comparisons for proportions tests at *P* = 0.05 [28]. For the same experiment, a factorial ANOVA was conducted with the ‘growth’ or ‘weight’ of shoots as dependent variables and ‘treatment’, ‘inoculation’ and their interaction as independent variables. Regarding the experiment with root exudates, two one-way ANOVAs were conducted with ‘MSG’ or ‘CG’ as dependent variables and ‘root exudates from treated plants’ as the independent variable. Depending on the levels of the independent variables and their interactions that were significant, the means of the treatments were compared using Fisher′s protected LSD test (*n* < 7) or Tukey′s HSD (*n* ≥ 7) (*P* = 0.05 in all cases) [27], with the exception of the effect of exposure periods on MS viability in each treatment, which was compared using orthogonal contrasts. Pearson correlation coefficients (*r*) between the effects of treatments on MS viability, disease development and/or root exudates were also estimated for the evaluated treatments (*n* = 6) using the average of MSG at 14 days of exposure, disease-related parameters (DI, Mortality, RDS and RAUDPC), and CG and MSG, respectively. Data from this study were analysed using Statistix 10.0 software [29].

## 3. Results

### 3.1. Viability of Verticillium dahliae Microsclerotia

**Figure 1:**
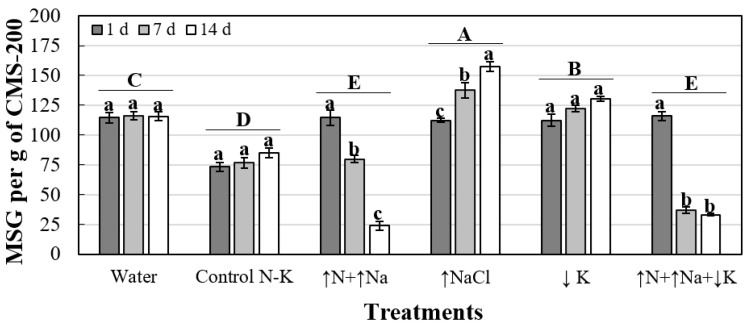
Effects of treatment and exposure period (1, 7 or 14 days) on microsclerotia germination of *V. dahliae* isolate V323 (MSG; number of germinated MS per g of CMS-200). For each treatment and exposure period combination, columns represent the means of MSG values of three replicated samples of CMS-200. For each treatment, columns with a common lowercase letter do not differ significantly according to orthogonal contrasts. Treatments with a common capital letter do not differ significantly according to Fisher’s protected LSD test at *P* = 0.05 for MSG at 14 days of exposure. Vertical bars represent the standard errors of the mean.

The effect of treatments on the viability of MS varied depending on the different combinations of treatments and exposure periods. There were significant differences between treatments, exposure periods and their interaction (*P* < 0.0001 in all cases). Therefore, the effect of the exposure periods on MS viability was analysed separately for each treatment using orthogonal contrasts (Figure 1). The effect of treatments on MS viability was also analysed separately for each exposure period (Table 2), although the ANOVA of the 14-day period was selected as the most representative of the three evaluated periods (Figure 1). 

Regarding the effect of the exposure periods on MS viability for each treatment, no significant differences in MSG between the exposure periods were observed for the Water, Control N-K and ↓K treatments. However, there was a significant increase in MSG over time of exposure for the ↑NaCl treatment and a significant decrease in MSG over time of exposure for the ↑N+↑Na and ↑N+↑Na+↓K treatments (Figure 1).

Concerning the effect of treatments on MSG at 14 days of exposure, the ↑NaCl treatment showed the highest MSG (157.6 ± 4.2 MS per g of CMS-200), followed by ↓K (130.6 ± 2.03 MS per g of CMS-200), whereas the ↑N+↑Na (23.7 ± 3.54 MS per g of CMS-200) and ↑N+↑Na+↓K (33.1 ± 1.1 MS per g of CMS-200) treatments showed the lowest MSG (Table 2).

### 3.2. Verticillium Wilt Development

Symptoms of Verticillium wilt disease were only observed in inoculated plants; therefore, non-inoculated plants were excluded from this analysis. In inoculated plants, disease symptoms developed from 6 weeks to 16 weeks after inoculation, after which disease progress was stopped. All disease-related parameters analysed in the inoculated plants showed significant differences (*P* < 0.05) between treatments (Table 3). Plants treated with ↓K showed the highest RAUDPC values (147.0 ± 11.9%), followed by those treated with ↑NaCl (119.6 ± 9.0%), with both values being significantly higher than those observed in the plants under the Control N-K treatment. However, plants treated with ↑N+↑Na or ↑N+↑Na+↓K showed significantly lower RAUDPC values than those observed in the plants under the Control N-K treatment. A similar pattern was observed for RDS since plants treated with ↑NaCl showed the highest RDS values (122.7 ± 27.4%), followed by those treated with ↓K (100.0 ± 4.6%). In this case, plants treated with ↑N+↑Na or ↑N+↑Na+↓K showed higher, but not significantly different, values of RDS than those observed in the plants under the Control N-K treatment. Conversely, plants treated with ↓K showed the highest incidence (75.0%), while in the remaining treatments, the results were similar to that observed in the plants under the Control N-K treatment. Regarding plant mortality, no significant differences between treated plants were observed, with ↑NaCl showing the lowest mortality throughout the experiment (8.3%) (Table 3).

There were no significant linear correlations (r < 0.8113, *P* > 0.05) between the disease-related parameters and MSG at 14 days of exposure.

**Figure 2:**
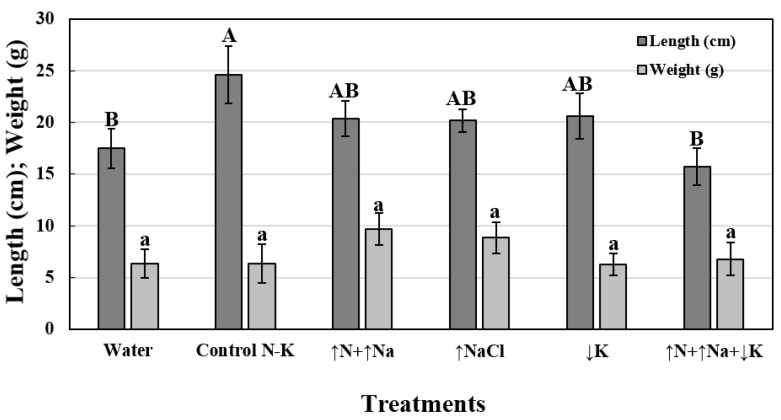
Effect of treatments on apical shoot development (length, cm; weight, g) in olive plants. For each dependent variable and treatment, columns represent the mean values of 24 plants, including both inoculated and non-inoculated plants, since no significant differences were found for the inoculation variable or its interaction with the treatments. Columns with the same uppercase or lowercase letter do not differ significantly according to Fisher’s protected LSD test (*P* = 0.05) for ‘length’ and ‘weight’ variables, respectively. Vertical bars represent the standard errors of the mean.

Regarding the effect of treatments on shoot growth, significant differences were observed only for the independent variable ‘treatments’ (*P* = 0.0280) and not for the independent variable ‘inoculation’ (*P* = 0.1160) or for the interaction ‘treatments’ × ‘inoculation’ (*P* = 0.0629). Therefore, values of ‘growth’ from both inoculated and non-inoculated plants were averaged for each treatment. To conduct further analysis, plants treated with water (17.5 ± 1.9 cm) or ↑N+↑Na+↓K (15.7 ± 1.8 cm) showed significantly lower increases in apical shoot length than those treated with the remaining treatments (↑N+↑Na = 20.4 ± 1.7; ↑NaCl = 20.2 ± 1.1; ↓K = 20.6 ± 2.2; Control N-K = 24.6 ± 2.8 cm) (Figure 2). Concerning the effect of treatments on the weight of the apical shoots, the factorial ANOVA did not show significant differences for either of the independent variables tested, i.e., ‘treatments’ (*P* = 0.2493) or ‘inoculation’ (*P* = 0.5726), but it did show significant differences for their interaction (*P* = 0.0187). However, all the treated means were grouped in a single group when they were compared by Tukey′s HSD test according to the level of the interaction (*n* = 12). Therefore, data were represented assuming the mean values of both lots of inoculated and non-inoculated plants for each treatment. Apical shoot weight values ranged between 9.7 ± 1.6 and 6.3 ± 1.0 g for plants treated with ↑N+↑Na and ↓K, respectively (Figure 2).

Finally, *V. dahliae* was consistently re-isolated from the stems of inoculated plants, with frequencies of isolation ranging from 57.1% to 85.7%. However, the pathogen was not re-isolated from non-inoculated plants. This fact confirmed that plants used for the experiment were initially healthy and indicated the success of the inoculation.

### 3.3. Effects of Root Exudates Collected from Non-Inoculated Plants Grown under Different Treatments

#### 3.3.1. Viability of *Verticillium dahliae* Microsclerotia

In the presence of root exudates from plants treated with water, the MSG was significantly increased (40.5 ± 0.5 MS per g of soil) compared to CaSO_4_ without root exudates (36.3 ± 1.0 MS per g of soil). However, the MSG was significantly reduced under the presence of root exudates from plants subjected to the different nutrient treatments compared to CaSO_4_ without exudates, with the exception of the ↑NaCl treatment (33.2 ± 1.6 MS per g of soil) (Table 4).

#### 3.3.2. Viability of *Verticillium dahliae* Conidia

In the presence of root exudates from plants treated with Control N-K, ↑NaCl, and ↑N+↑Na+↓K, the CG was increased significantly compared to CaSO_4_ without exudates. Conversely, significantly lower values of GC corresponding to the Water, ↑N+↑Na, and ↓K treatments were detected (Table 4).

Finally, there were no significant linear correlations between the effect of root exudates from treated plants on the CG and MSG (r = −0.1037, *P* = 0.8450) as well as between these two parameters and the MSG at 14 days of exposure or any of the disease-related parameters (r < 0.5447, *P* > 0.05).

## 4. Discussion

The effect of mineral nutrition on wilt diseases has already been reported in a wide diversity of herbaceous hosts/Verticillium pathosystems [1]. In this regard, it is generally understood that N excess or K deficiency tends to increase or decrease, respectively, the incidence of plant diseases, including Verticillium wilts [1,11]. However, other aspects such as stage of plant growth or pathogen activity, form of the elements (reduced or oxidized forms), soil conditions and interactions with other elements can influence plant response against diseases [1,9]. In fact, several authors have reported that high levels of N increased *V. dahliae* susceptibility in aubergine, cotton, potato and tomato, whereas others showed opposite results in these same hosts, i.e., low levels of N increased the susceptibility and high levels of N reduced it [1]. Concerning the effect of K deficiency on plant diseases, it is well documented that plants under K starvation are more susceptible than plants under optimum K nutrition, indicating that K affects host resistance more than it directly affects the pathogen [1,11]. However, the influence of mineral nutrition on VWO is still poorly studied. Despite the fact that several field observations conducted in southern Spain reveal that N excess or, more probably, imbalances of N excess and K deficiency could be related to a higher incidence and severity of the disease in the affected olive groves, this phenomenon has not yet been demonstrated. Therefore, the present work was conceived to shed light on the effects of nutritional imbalances of N and K on the infection of *V. dahliae* in olive trees. 

In this study, an *in vitro* test was first conducted to determine both the effects of N-K imbalances and the influence of the exposure period to each treatment on the MSG of *V. dahliae*. Considering the 14-day exposure period, ↑NaCl and ↓K increased the MSG as the exposure period increased; whereas ↑N+↑Na and ↑N+↑Na+↓K significantly reduced the MSG compared to the Water and Control N-K treatments, the effect of N being predominant compared to that of Na in all cases.

Considering the effect of N *in planta*, plants subjected to N excess or N-K imbalances showed lower RAUDPC compared to both Control N-K and Water treatments, with only significant differences for N excess. However, non-significant differences between them were observed for RDS. The effect of N excess on VWO was not only unclear, but also opposite to our previous hypothesis. Therefore, the reduction of MSG of *V. dahliae* and the lower values of RAUDPC in olive plants treated with ↑N+↑Na or ↑N+↑Na+↓K under our experimental conditions may have been greatly influenced by the N forms used in this study. Indeed, direct effects of N ions (e.g., NO_3_^−^, NH_4_^+^) have been reported by reducing the number of the propagules of *V. dahliae* [1]. 

The effect of K deficiency in increasing MSG was in concordance with the effect observed *in planta* since plants subjected to this treatment showed significantly higher RAUDPC and RDS compared to the Control N-K and Water treatments. Accordingly, previous studies conducted in cotton demonstrated that susceptible cultivars to *V. dahliae* showed lower tolerance to wilt when they were grown in K-deficient soils [1]. Thus, the effect of K-fertilization in reducing the severity of wilt diseases has only been considered beneficial in K-deficient soils [11]. Moreover, it has been demonstrated that K controls many physiological and metabolic processes and acts as an enzyme activator, enhancing several signalling cascades similar to biotic stress responses, which could affect host resistance against pathogens [1,11,12,13,14,30]. Regarding this last aspect, plants subjected to K starvation induce H_2_O_2_, a reactive oxygen species, increasing the salicylic acid (SA) levels and the biosynthesis of jasmonic acid (JA), rapidly increasing the levels of this hormone [13]. Altogether, these data indicate that K deficiency negatively influences plant tolerance against diseases since the plant response against K starvation involves inducing the signal transduction pathways against environmental stresses, i.e., SA and JA pathways. However, although both SA and JA mediate the activation of plant defence pathways [31], this host defence response is likely not enough to prevent *V. dahliae* infections in olive when induced as a consequence of K deficiency.

Concerning the N-K imbalances, notice that the effect of N prevails rather than that of K since the treatments with N excess or N-K imbalances decrease MSG or the disease progress, whereas treatments with K deficiency alone increase both parameters. Interestingly, in spite of the Na excess treatment that was included as a technical control in this study, a similar behaviour to that observed for K deficiency was shown, i.e., the MSG and RAUDPC were higher compared to the Control N-K treatment. These observations are probably due to the phytotoxic effect that NaCl excess has *per se* or because NaCl induces K deficiency, affecting the host resistance, as described previously [13,30]. 

The non-significant effect on plant growth development between treatments could be due to the short periods that the plants were subjected to the treatments under our experimental conditions. Although more time would be needed to evaluate the effect of mineral nutrition on plant growth, the experimental procedure in this study took into account that the experiments must concluded when the disease reaches the maximum levels of severity, making it impossible to extend the evaluations. Moreover, the non-significant differences on plant weight between inoculated and non-inoculated plants was probably due to the fact that the wilt symptoms developed late, at the end of the experiment, though the disease progressed rapidly afterwards.

Finally, the study on the effect of root exudates collected from the treated plants on the viability of conidia and MS of *V. dahliae* was conducted here because little information is currently available in the literature on this topic. Our results indicate that root exudates from treated plants reduced the germination of MS compared to those from plants treated with only water, but they generally increased the germination of conidia. Recently, López-Moral et al. [26] demonstrated that root exudates from olive of cv. Picual significantly induced the germination of conidia and MS of *V. dahliae* compared to the control without root exudates. Thus, our results suggest that nutritional treatments can have an indirect effect on pathogen infection by reducing the ability of root exudates to stimulate the germination of MS of *V. dahliae*. 

Considering all our results together, we can confirm that disease development depends on factors that may affect the plant, the pathogen or their interaction. These effects may also act at different stages of the life cycle of the pathogen, such as in the germination of MS, the infection process and colonization of the plant or enhancing the mechanisms of plant defence. In this complex system, nutrient inputs can have an effect on different processes, and it would be interesting to learn more about these. Nevertheless, little attention has been given to understanding the relationships between K and other nutrients such as N in relation to plant diseases and to understanding the mechanisms by which N and K influence diseases. Likewise, this work attempts to provide new knowledge, studying not only the effect of mineral nutrition on disease development, but also their direct effect on the propagules of *V. dahliae* and the indirect effect through possible modifications in root exudates affecting the first stages of the infection. However, we must remember to consider the conclusions of this study as a first approach, opening up new paths to be explored towards a better understanding of how mineral nutrition affects VWO development, e.g., comparing both NO_3_^−^ and NH_4_^+^ forms of N, even more K-deficient treatments, the effect of NaCl, and longer exposure to treatments. Altogether, these findings will generate interesting knowledge in applied plant pathology and agronomy sciences within the framework of integrated disease management in terms of cultural practices towards the control of VWO through mineral nutrition management.

## 5. Conclusions

N excess or N-K imbalances decrease the germination of *V. dahliae* MS or the disease progression in olive plants inoculated with the pathogen, whereas K deficiency increases both parameters. Therefore, the effect of N excess on MS germination or on the disease progress predominates rather than the effect of K deficiency. Although Na excess was included as a technical control in this study, a similar behaviour to that noted for K deficiency was observed. Finally, root exudates from treated plants induced conidia germination compared to the Control N-K treatment but reduced the MS germination. This last fact could be due to an indirect effect caused by the mineral nutrition state in the plant, which should be studied more closely in the future. This study reveals that balanced nutrition must be considered as an important factor in plant resistance to diseases, opening up new paths to be explored towards a better understanding of how mineral nutrition affects VWO development, such as comparing both NO_3_^−^ and NH_4_^+^ forms of N, even more K deficiency treatments, the effect of NaCl, and longer exposure to treatments.

## Figures and Tables

**Table 1:** Composition of the treatments used in this study.

Treatment	Compound [mM]	Element [mM]
Control N-K	Ca(NO_3_)_2_ [1.5] KNO_3_ [2.0]	N [5.0] K [2.0]
N and Na excess (↑N+↑Na)	Ca(NO_3_)_2_ [1.5] KNO_3_ [2.0] NaNO_3_ [45.0]	N [50.0] K [2.0] Na [45.0]
NaCl excess (↑NaCl)	Ca(NO_3_)_2_ [1.5] KNO_3_[2.0] NaCl [45.0]	N [5.0] K [2.0] Na [45.0]
K deficiency (↓K)	Ca(NO_3_)_2_ [1.5] NaNO_3_ [2.0]	N [5.0] Na [2.0]
N and Na excess and K deficiency (↑N+↑Na+↓K)	Ca(NO_3_)_2_ [1.5] NaNO_3_ [47.0]	N [50.0] Na [47.0]

**Table 2:** Effects of treatments on microsclerotia (MS) viability of *V. dahliae* isolate V323 for each exposure period (1, 7 and 14 days) evaluated.

Treatment	MSG (MS Per g of CMS-200) ^a^		
	1 Day	7 Days	14 Days
Water	114.8 ± 4.4 a	116.3 ± 3.6 b	115.7 ± 3.6 c
Control N-K	73.2 ± 3.9 b	76.5 ± 4.5 c	85.2 ± 3.9 d
↑N+↑Na	114.5 ± 6.2 a	79.9 ± 3.1 c	23.7 ± 3.5 e
↑NaCl	112.4 ± 1.6 a	137.7 ± 6.4 a	157.6 ± 4.2 a
↓K	112.4 ± 5.1 a	122.2 ± 2.5 b	130.6 ± 2.0 b
↑N+↑Na+↓K	116.1 ± 3.8 a	36.8 ± 2.7 d	33.1 ± 1.1 e
*P*-value _(α = 0.05)_	0.0004	≤0.0001	≤0.0001

^a^ MSG: Number of germinated MS per g of CMS-200 after 1, 7 or 14 days of exposure to each treatment (MS per g of CMS-200). For each treatment and exposure period combination, values represent the means of three replicated samples of CMS-200 ± standard errors of the mean. In each column, means followed by a common letter do not differ significantly according to Fisher′s protected LSD test (*P* = 0.05).

**Table 3:** Disease-related parameters for olive plants treated and inoculated with *V. dahliae* isolate V323 ^a^.

Treatment	Incidence (%) ^b^	Mortality (%) ^b^	RDS (%) ^c,e^	RAUDPC (%) ^d,e^
Control N-K	58.3 ab	16.7 ab	69.3 ± 5.3 b	108.7 ± 14.7 b
↑N+↑Na	58.3 ab	25.0 a	80.0 ± 12.2 ab	63.4 ± 7.2 c
↑NaCl	66.7 ab	8.3 b	122.7 ± 27.4 a	119.6 ± 9.0 ab
↓K	75.0 a	25.0 a	100.0 ± 4.6 ab	147.0 ± 11.9 a
↑N+↑Na+↓K	50.0 b	25.0 a	85.3 ± 14.1 ab	89.9 ± 5.9 bc
Water (+) ^f^	66.7 ab	16.7 ab	100.0 ± 10.6 ab	100.0 ± 22.4 b
Water (−) ^g^	0.0 c	0.0 c	0.0 ± 0.0 c	0.0 ± 0.0 d
*P*-value _(α = 0.05)_	≤0.0001	≤0.0001	0.0014	≤0.0001

^a^ Healthy olive plants were grown in a substrate infested with a sterile cornmeal–sand mixture (CMS; sand, cornmeal and distilled water; 9:1:2, *w*:*w*:*v*) colonized by *V. dahliae* isolate V323 and adjusted at a final theoretical inoculum density of 10^7^ CFU g^−1^. Disease-related parameters were assessed weekly for 16 weeks after inoculation with *V. dahliae*. ^b^ Percentage of symptomatic plants (Incidence) or dead plants (Mortality) at 16 weeks after planting in the infested substrate with *V. dahliae* isolate V323 (*n* = 24). In each column, mean values followed by a common letter do not differ significantly according to the multiple comparisons for proportions test [28] at α = 0.05. ^c^ RDS: Relative disease severity. ^d^ RAUDPC: relative area under the disease progress curve. ^e^ RDS, RAUDPC estimated as the relative percentage (%) to disease parameter values of the inoculated plants treated with only DDW (Water (+)) at the end of the experiment. Mean values represent the average of two experiments with 3 blocks and 4 replicated plants per block for each treatment ± standard error of the mean. In each column, mean values followed by a common letter do not differ significantly according to Fisher’s protected LSD test (*P* = 0.05). ^f^ Water (+): non-treated and inoculated plants. ^g^ Water (−): non-treated and non-inoculated plants.

**Table 4:** Effects of root exudates ^a^ from non-inoculated plants on conidia germination of *V. dahliae* isolate V323 and microsclerotia viability in naturally infested soil samples.

Treatments	CG (%) ^b,d^	MSG (MS Per g of Soil ^c,d^)
Water	83.0 ± 1.0 c	40.5 ± 0.5 a
Control N-K	88.0 ± 0.6 b	32.5 ± 1.6 c
↑N+↑Na	85.8 ± 2.3 bc	27.9 ± 0.3 d
↑NaCl	93.5 ± 2.4 a	33.2 ± 1.6 bc
↓K	82.4 ± 0.6 c	28.4 ± 1.1 d
↑N+↑Na+↓K	88.0 ± 0.8 b	25.9 ± 0.9 d
CaSO_4_ (−) ^e^	81.2 ± 1.9 c	36.3 ± 1.0 b
*P*-value _(α = 0.05)_	0.0021	0.0003

^a^ Root exudates were collected by dipping the plant roots in 0.01 M CaSO_4_ solution for 4 h. ^b^ CG: percentage (%) of germinated conidia after incubation in the root exudates at 24 °C for 24 h in the dark. Values represent the means of two experiments with five replicate cover slides each ± standard errors of the mean. ^c^ MSG: Number of germinated MS per g soil after 72 h of exposure to the root exudates. Values represent the means of two experiments with three replicate soil subsamples each ± standard errors of the mean. ^d^ In each column, means followed by the same letter do not differ significantly according to Fisher’s LSD test (*P* < 0.05). ^e^ CaSO_4_ (−): CaSO_4_ solution (0.01 M) without root exudates was used as the negative control in this experiment.

## Data Availability

The data that support the findings of this study are available from the corresponding author upon reasonable request.
